# Purification and characterization of the first recombinant bird pancreatic lipase expressed in *Pichia pastoris*: The turkey

**DOI:** 10.1186/1476-511X-10-24

**Published:** 2011-01-27

**Authors:** Madiha Bou Ali, Yassine Ben Ali, Aida Karray, Ahmed Fendri, Youssef Gargouri

**Affiliations:** 1 Laboratoire de Biochimie et de Génie Enzymatique des Lipases, ENIS route de Soukra, BP1173, University of Sfax, 3038 Sfax, Tunisia

## Abstract

**Background:**

The turkey pancreatic lipase (TPL) was purified from delipidated pancreases. Some biochemical properties and kinetic studies were determined using emulsified system and monomolecular film techniques. Those studies have shown that despite the accumulation of free fatty acids at the olive oil/water interface, TPL continues to hydrolyse efficiently the olive oil and the TC_4 _in the absence of colipase and bile salts, contrary to most classical digestive lipases which denaturate rapidly under the same conditions. The aim of the present study was to express TPL in the methylotrophic yeast *Pichia pastoris *in order to get a large amount of this enzyme exhibiting interesting biochemical properties, to purify and characterize the recombinant enzyme.

**Results:**

The recombinant TPL was secreted into the culture medium and the expression level reached about 15 mg/l after 4 days of culture. Using Q-PCR, the number of expression cassette integrated on *Pichia *genomic DNA was estimated to 5. The purified rTPL, with molecular mass of 50 kDa, has a specific activity of 5300 U/mg on emulsified olive oil and 9500 U/mg on tributyrin. The optimal temperature and pH of rTPL were 37°C and pH 8.5. The stability, reaction kinetics and effects of calcium ions and bile salts were also determined.

**Conclusions:**

Our results show that the expressed TPL have the same properties as the native TPL previously purified. This result allows us the use of the recombinant enzyme to investigate the TPL structure-function relationships.

## Background

Lipases are defined as triacylglycerol acylhydrolases (E.C.3.1.1.3) that catalyze the hydrolysis of fats and oils at the oil-water interface to glycerol and free fatty acids. Although lipases belong to many different protein families without sequence similarity, there is a much greater conservation in the secondary and tertiary structures of lipases [1].

Over the last years, a better understanding of pancreatic lipases structure-function relationships has been reached with the resolution of several three-dimensional structures [2,3]. The resolution of the three-dimensional structure of human pancreatic lipase (HPL) [2] has shown that the single polypeptide chain (449 amino acids) is fold into two domains: a large N-terminal domain (residue 1-335) which shows a typical α/β hydrolase fold and a small C-terminal domain (residue 336-449) which is of β sandwich type [4].

The N-terminal domain contains the active site, which involves a catalytic triad analogous to that present in serine proteases. A surface loop (Cys 237-Cys 261), the so-called lid or flap, prevents the access of the substrate to the active site in its closed conformation. HPL requires a small protein cofactor, colipase, for the enzyme to be able to bind, in the presence of bile salt, to the triacylglycerol/water interface. Colipase binds to the C-terminal domain of HPL and exposes the hydrophobic tips of its fingers at the opposite side of its lipase-binding site [5]. The open lid and the extremities of the colipase fingers, as well as the β9 loop, form an impressive continuous hydrophobic plateau extending over more than 50 A°^2^, which might be to interact strongly with a lipid/water interface [6].

The turkey pancreatic lipase (TPL) was purified from delipidated pancreases. This avian pancreatic lipase contains 450 amino acids and presents an experimental mass of 49665.31 Da [7]. Some biochemical properties and kinetic studies were determined using emulsified system and monomolecular film techniques [8,9]. Those studies have shown that despite the accumulation of free fatty acids at the olive oil/water interface, TPL continues to hydrolyse efficiently the olive oil and the TC_4 _in the absence of colipase and bile salts, contrary to most classical digestive lipases which denaturate rapidly under the same conditions [10,11].

Fendri et al. have determined the critical surface pressure (Πc) of TPL corresponding to the interaction power with Egg-PC monolayers. They found that TPL presents a higher Πc (Πc = 29 mNm^-1^) [9] in comparison with HPL (Πc = 18 mNm^-1^) [11]. This result has been explained by the fact that the hydrophobic surface of TPL is higher by 166 A°^2^, as compared to that of HPL. So, hydrophobic interactions between the lipase and the substrate would be more efficient in the case of TPL than in that of HPL.

The cleavage of TPL by chymotrypsin has generated three major fragments of about 35, 14 and 10 kDa. The N-terminal of the 35 kDa fragment was the same as the native TPL [12]. So this truncated TPL form would correspond to the TPL N-terminal domain which was active on tributyrin emulsion in the absence of colipase and in the presence of a low concentration of bile salts. Others attempts to produce an active N-terminal pancreatic lipase domain by limited proteolysis were failures with the HPL, PPL and OPL [12-14], except with the horse pancreatic lipase giving to a large N-terminal fragment (45 kDa) which retained the lipase activity.

To investigate the structure-fonction relationships of the TPL, a high expression level system of TPL is required. The methylotrophic yeast *Pichia pastoris *is a host system which has been widely used at both academic and industrial laboratories in the production of a variety of heterologous proteins [15].

*Pichia pastoris *has a strong preference for respiratory growth, consequently it doesn't produce significant amount of toxic ethanol as compared to many species of the genus *saccharomyces *[16] and may grow to very high cell densities on inexpensive media.

Like other eukaryotic expression systems, *Pichia *pastoris presents many advantages, such as proteolytic processing, folding, disulphide bonds formation and glycosylation, as well as several other post-translational modifications.

This expression system gives efficient recombinant protein secretion level which, combined with the very low endogenous protein secretion level, is a great advantage for the purification of the recombinant protein secreted into the culture medium [15]. *Pichia pastoris *system based on the constitutive promoter glyceraldehyde-3-phosphate dehydrogenase gene (GAP) have recently became available [17]. The GAP promoter for the heterologous expression of genes giving products which are not toxic to *Pichia pastoris*, or when the use of methanol, a highly toxic solvent, has to be ruled out.

In this paper, we report the expression of the TPL gene in *Pichia pastoris*, the recombinant enzyme was purified and its properties are compared to the native one.

## Results and discussion

### Generation of recombinant *Pichia *clones expressing TPL

The TPL gene, containing two EcoRI sites upstream of its first codon and downstream of its Stop codon, was digested by the EcoRI restriction enzyme and inserted into the pGAPZαA vector previously digested by EcoRI. The recombinant vector was then linearized by the BspHI restriction enzyme and the GAP promoter-driven constitutive expression of TPL was achieved by integrating the linearised pGAPZαA-TPL plasmid into the X33 wild type *P-Pastoris *genome at the GAP locus. *P.pastoris *tranformants generated by electroporation with the recombinant vector (pGAPZαA-TPL) were selected on YPDS plates containing Zeocin and incubated at 30°c for 3 days. Six randomly picked Zeocin resistant positives clones on the solid selective medium were selected and the presence of TPL insert in the Zeo-resistant transformants was checked by PCR.

### Lipase producing level of selected clones

**Figure 1:**
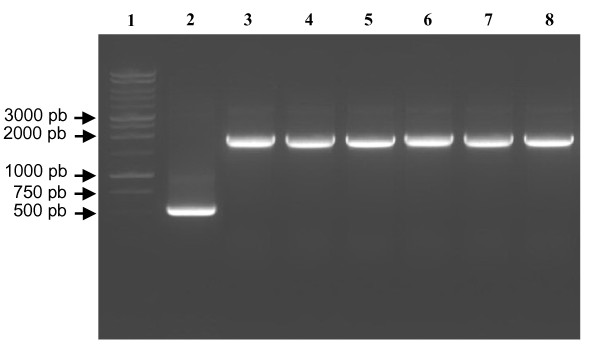
**Analysis by electrophoresis performed on 1% agarose gel of PCR products**. Lane 1: 1 kb Ladder; lane 2: amplified products obtained using pGAPZαA vector as template; Lane 3, 4, 5, 6,7 and 8: amplified products obtained using as template the genomic DNA extracted from clones transformed with the vector pGAPZαA/TPL (C1, C2, C3, C4, C5, C6).

**Figure 2:**
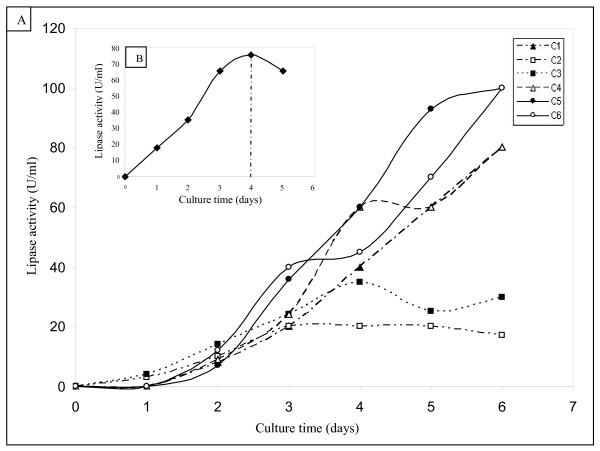
**Time-course of rTPL expression.**  (A) The six isolated clones of recombinant *P.pastoris*. (B, insert) The time-course of the rTPL expression by the selected clone (C5).

To confirm the integration of the TPL gene into the *P.pastoris *genome, we have performed a PCR reaction using as template the genomic DNA of the selected clones. We found that the C1, C2, C3, C4, C5 and C6 clones, show an amplification of a 1900 pb fragment corresponding to the TPL gene (1353 pb) plus a portion of the vector (540 pb) which is amplified even in the absence of integration (Figure 1). This result suggests the integration of the TPL DNA into the *Pichia *X33 genome (Figure 1). Positive clones were then cultivated in Erlenmeyer's flasks to identify which clone has the highest lipase production level. Samples were collected during 6 days and assayed for the lipase activity (Figure 2A).

Figure 2A describe the variation of rTPL activity in the culture medium as function of culture days. It shows that the C5 and C6 clones exhibit the highest activity levels which reach about 100 U/ml after 6 days of culture. C5 clone was chosen to continue this study.

It is well known that protease degradation is the main problem of protein expressed in heterologous system [18]. One solution to avoid this degradation is to reduce the culture period. In this purpose we have chosen to start the cell culture with an OD of 1, which allowed a shift of the production period from 6 to 4 days (Figure 2B).

The secretion of the recombinant protein was detected 24 h after the culture was initiated, and a maximum activity level of 75 U/ml was reached after 4 days. Starting the 5^th ^day, a slight decrease in the rTPL activity was observed suggesting a probable proteolytic degradation of the expressed protein.

### Analysis of TPL cDNA copies number in selected recombinant strains by Q-PCR

**Figure 3:**
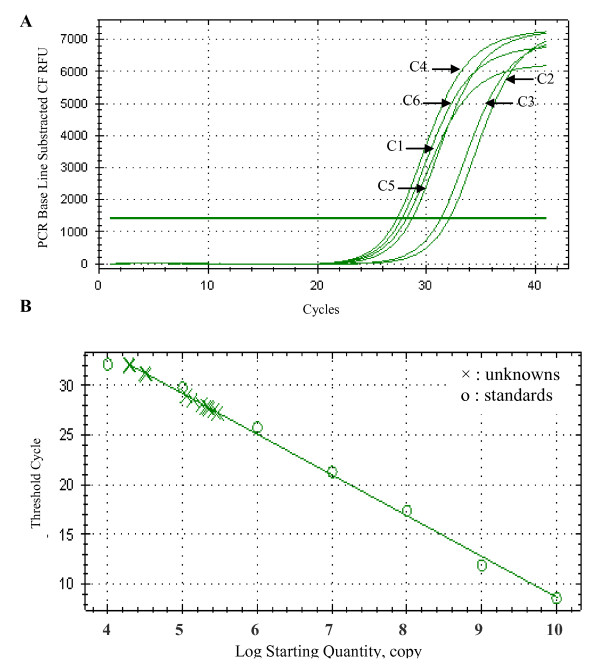
**Q-PCR analysis of the TPL cDNA copies number in the genomic DNA of C1, C2, C3, C4, C5 and C6 clones**. Correlation coefficient: 0.993; Slope: -4.100; PCR Efficiency: 75.3%

Genomic DNA extracted from the six selected clones were analyzed by Q-PCR to estimate the TPL cDNA copies number. As shown in figure 3A, C1, C4, C5 and C6 clones have approximately the same Ct = 28, reflecting an identical copies number of TPL cDNA in their genomic DNA. The C2 and C3 clones have a Ct value of 31.

Based on the standard curve done with 10 fold serial dilution of plasmid DNA (pGAPZαA/TPL), the number of expression cassette integrated was estimated to five copies for C1, C4, C5 and C6 and four copies for C2 and C3 clones (Figure 3B). These results are in agreement with the difference in the activity levels of these clones (Figure 2A). In fact C2 and C3 exhibit the lowest lipase activity level compared to the other clones.

### Production of rTPL in *P.pastoris*

After 4 days of yeast growth, the corresponding amount of recombinant TPL reached about 15mg/l of culture medium. This production rate can be middling in comparison with other production levels of various esterases and lipases expressed in *P.pastoris*. For comparison, the production rate of the Carp acetylcholinesterase is about 40 μg/l of culture medium after 10 days of methanol induction [19]. The human pancreatic lipase-related protein 2 has a productivity of 40 mg/l under the control of the AOX1 promoter and about 4 mg/l under the control of the constitutive GAP promoter [20]. This difference could not be caused by the constitutive promoter efficiency because this promoter has been successfully used to secrete a high level of mature rHPLRP1 (100-120 mg/l) [21]. Recombinant human Bile salt-simulated lipase has a production amount of 0.8-1 g/l [22].

Some studies described the expression of fungical lipases in *P.pastoris*, the Lip2 lipase of *Yarrowia lipolytica *was successfully expressed in *P.pastoris *with an expression level of around 0.63 g/l [23]. *C.rugosa *Lip4 lipase [24] and *Mutant Nippostrogglus brasiliensis *Acetylcholinesterase [25] were expressed with a production level of 0.1 and 2 g/l respectively.

### Purification of rTPL expressed in *P.pastoris*

After 4 days of time course fermentation, 500 ml of culture broth were centrifuged at 9500 rpm for 30 min to discard the yeast cells.

### Ammonium sulfate precipitation

The supernatant was brought to 60% saturation with solid ammonium sulfate under stirring conditions and maintained for 60 min at 4°C. After centrifugation (30 min at 9500 rpm), the precipitate was resuspended in 5 ml of buffer A (250 mM Tris-HCl, pH 8.2, 250 mM NaCl, 20 mM Benzamidine). Insoluble material was removed by centrifugation for 10 min at 9500 rpm.

### Anion exchange chromatography on DEAE -Cellulose

The supernatant was diluted 10x with distilled water and loaded on a DEAE-Cellulose anion exchanger equilibrate in buffer B (25 mM Tris-HCl pH 8.2, 25 mM NaCl, 2 mM Benzamindine). Under these conditions, the rTPL doesn't absorb on the anionic support and was then eluted during the washing step using the same buffer.

### Filtration on Sephacryl S200

**Figure 4:**
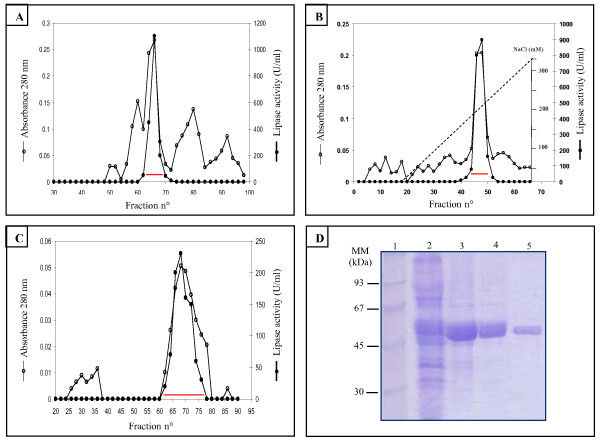
**Purification steps of rTPL**. (A) Chromatography of rTPL on Sephacryl S200; the column (2.5 × 150 cm) was equilibrated with 25 mM Tris buffer, pH 8.2, containing 25 mM NaCl and 2 mM Benzamidine (buffer B). The elution of proteins was performed with the same buffer at a rate of 30 ml/h. **(B) **Chromatography of rTPL on FPLC Mono-Q Sepharose. The column was equilibrated with buffer B; a linear gradient was applied from 25 to 350 mM NaCl in buffer B; the flow rate used was 2 ml/min. **(C) **Chromatography of rTPL on Sephadex G100; the column was equilibrated in buffer B and the flow rate was 25 ml/h. The pooled fractions containing the rTPL activity were indicated by a dashed line. **(D) **SDS-PAGE analysis of rTPL performed on 12% gel. Lane1: Low molecular weight marker; Lane 2: culture supernatant; Lane 3: active fraction from Sephacryl-S200; Lane 4: active fraction from Q-Sepharose FF; Lane 5: active fraction from Sephadex-G100.

Active fractions were pooled and concentrated using an Amicon Ultra-15 (molecular weight cutoff of 10 kDa). The concentrate was applied on a column of gel filtration Sephacryl S-200 equilibrated with buffer B. Proteins elution was performed with buffer B at a flow rate of 30 ml/h. rTPL was eluted between 1.3 and 1.5 void volumes (Figure 4A).

### Anion exchange chromatography Q-Sepharose FF

Active fractions eluted from Sephacryl S200 were pooled, concentrated then applied to FPLC equipped with a Q-Sepharose FF column equilibrated with buffer B. The column was washed with the same buffer, then, proteins were eluted with a linear NaCl gradient from 25 to 350 mM in buffer B. rTPL activity was eluted between 180 and 220 mM NaCl (Figure 4B).

### Filtration on Sephadex G100

Active fractions were pooled, concentrated then loaded on a second gel filtration Sephadex G-100 equilibrated with buffer B. Elution of rTPL was performed with buffer B at a flow rate of 25 ml/h. rTPL was eluted at 1.2 void volume (Figure 4C).

The fractions containing the rTPL activity were pooled and analyzed on SDS-PAGE.

A summary of the purification process is given in table 1. Starting with the whole *P.pastoris *culture supernatant, 13-fold purification was achieved and the overall recovery of enzyme activity was 32%. The purification resulted in a significant increase in the specific activity from 399 U/mg to 5300 U/mg.

**Table 1:** Flow sheet of rTPL purification

Purification step	Total activity (U)	Specific activity (U/mg)	Yield (%)	Fold
Supernatant	34380	399	100	1
(NH_4_)_2_SO_4 _Precipitation	30800	1294	89.6	3.24
DEAE-Cellulose	24650	1454	71.7	3.64
Sephacryl S-200	23540	3789	68.5	9.49
Q-Sepharose FF	14790	4150	43	10.4
Sephadex G-100	11000	5300	32	13.28

From 500 ml of *P.pastoris *cell culture media, about 2 mg of pure rTPL was obtained with a specific activity of 5300 U/mg using gum arabic emulsified olive oil as substrate in the presence of colipase and 4 mM NaDC at pH 8.5 and 37°C.

### SDS-PAGE

The fractions containing rTPL activity were pooled and analyzed on SDS-PAGE (Figure 4D). This figure shows that a single band with an apparent molecular weight of 50 kDa was revealed for the recombinant TPL.

### N-terminal Sequencing

**Figure 5:**
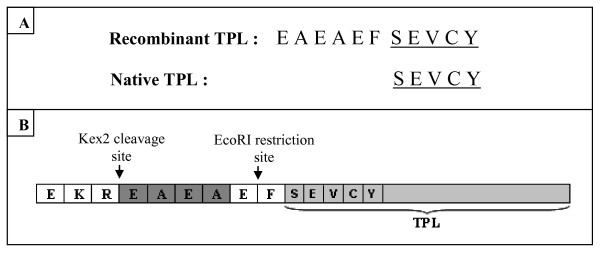
**Recombinant TPL N-terminal sequence.(A)** Alignment of native and recombinant TPL N-terminal sequences. **(B) **Amino acid sequences around the junction of signal peptide (α-factor) and mature TPL.

The N-terminal aminoacide sequence of recombinant TPL revealed 11 residues EAEAEF**SEVCY **(Figure 5A). This sequence shows the correct cleavage of the α-factor signal sequence at the Kex2 cleavage site. Compared with the native lipase, the N-terminal sequence of the recombinant lipase bears six amino acid residues derived from the EcoRI site (E and F residues) and residues separating the Kex2 cleavage site and the EcoRI site (E,A,E, and A residues) in the pGAPZαA vector (Figure 5B).

It is worth noticing that despite the presence an extra peptide of 6 amino acids on its N-terminal part, the rTPL seems to share the same biochemical and kinetic properties with the native enzyme under the same experimental conditions (pH stat technique). It will be interesting to compare the interfacial properties of both enzymes using a more sensitive method as the monomolecular film technique.

### Effect of temperature and pH on the activity and the stability of the rTPL

**Figure 6:**
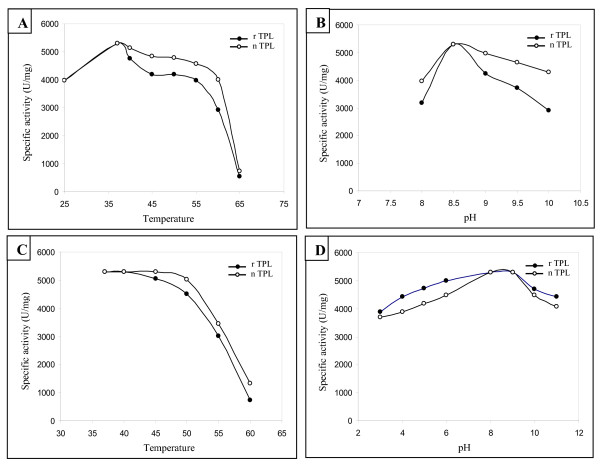
**Effects of temperature and pH on rTPL activity and stability. (A)** Effect of temperature on rTPL and nTPL activities. **(B) **Effect of pH on rTPL and native TPL activities. **(C) **Effect of temperature on rTPL and nTPL stability. The enzymes are incubated at different temperatures for 30 min. **(D) **Effect of pH on rTPL and nTPL stability. The enzymes are incubated at different pH for 30 min. Lipase activity was performed under standard conditions.

In order to check if the optimal pH and temperature of lipase activity of TPL was affected compared to native one, we measured at different pH and different temperatures the TPL activity (Figure 6A and 6B). The results show that both rTPL and nTPL present an optimum activity at 37°C and pH 8.5 (Figure 6A-6B). At 55°C, the rTPL and the nTPL keep 75% and 85% of their activity respectively. Up to 55°C, the two enzymes retain only 10% of their activities.

To study the thermal stability of the rTPL and the nTPL, each enzyme was incubated at various temperatures for 30 min at pH 8. The results show that both recombinant and native enzymes exhibit similar thermal stability behaviour. In fact, the two enzymes retain 80% and 95% of their activity, respectively, after 30 min of incubation at 50°C. At temperature over 55°C, the activity decreased dramatically to reach 15 to 20% for both enzymes at 60°C (Figure 6C).

For the enzyme stability at different pH, the measurement of residual activity shows that both enzymes are stable in a wide pH range. Indeed, the rTPL and native TPL keep 100% of their activity after 30 min incubation at pH 8 and 9. When incubated at pH 3, both enzymes retained 70% of their activities (Figure 6D).

### Effect of bile salts and calcium ions on the rTPL activity

It is well established that bile salts are strong inhibitors of all pure pancreatic lipases independently of their origins [26-28].

**Figure 7:**
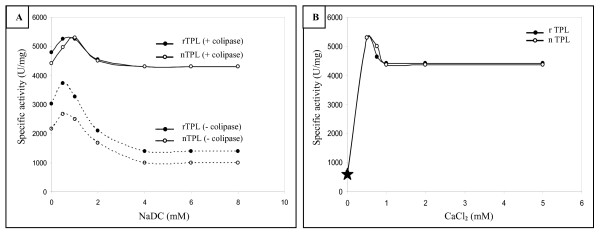
**Effect of increasing concentrations of bile salt (NaDC)  and calcium ions (Ca2+) on the rate of hydrolysis of olive oil emulsion by the rTPL. (A)** Effect of increasing concentration of bile salt (NaDC) on the rate of hydrolysis of olive oil emulsion by the rTPL and the nTPL. Lipolytic activity was measured at pH 8.5 and 37°C in the absence or in the presence of a molar excess of colipase. **(B) **Effect of increasing concentration of calcium ions (Ca^2+^) on the rate of hydrolysis of olive oil emulsion by the rTPL and the nTPL. Lipolytic activity was measured at pH 8.5 and 37°C. The star indicates the lipase activity measured in the absence of CaCl2 and in the presence of 10 mM EDTA.

The effect of varying concentrations of sodium deoxycholate (NaDC) on the rTPL and nTPL activities shows that, contrary to classical pancreatic lipases, the two enzymes are partially inhibited by the NaDC and retains respectively 30 and 25% of their activities at 8 mM NaDC (Figure 7A).

In order to investigate the calcium dependence of the rTPL and nTPL, we measured the hydrolysis rates of olive oil emulsion by the two enzymes in the presence of various CaCl_2 _concentrations. Our results showed that, unlike most classical pancreatic lipases [29], calcium is not necessary to trigger rTPL and nTPL activities (Figure 7B) and, in the absence of CaCl_2_, the rTPL and nTPL retained about 10% of their activities. The maximal TPL activity was measured at 0.5 mM CaCl_2_.

## Conclusion

In this study we reported the cloning of TPL gene into *P.pastoris *and the expression of the enzyme as a functional form. The expression level of the rTPL reached about 15 mg/l of culture medium after 4 days fermentation. The purified rTPL has a molecular mass of 50 kDa and a specific activity of 5300 U/mg on emulsified olive oil and 9500 U/mg on tributyrin substrate. Some previous studies showed that the heterologous expression could lead to a modification of same biochemical properties [30]. In our case, the biochemical characterization of the rTPL and its comparison to the native TPL shows that the rTPL seams to be identical to the native enzyme which makes *P.pastoris *a promising system for the expression of TPL and its eventual mutants. This statement is suitable for using rTPL instead of nTPL to study the structure-function relationships of this interesting enzyme.

## Materials and methods

### Yeast culture media

*Pichia pastoris *liquid cell cultures were grown in YPD medium containing 10 g yeast extract, 20 Bacto-peptone and 20 g D-glucose. The YPDS medium was YPD medium to which 18.2 g sorbitol per liter was added. To prepare plates for solid cell cultures, 2% agar (w/v) was added to the YPD medium.

### Strains, plasmid and reagents

The *P.pastoris *host strain was X33 (wild-type strain from Invitrogen). The *P.pastoris *transfer vector pGAPZαA (Invitrogen) used for yeast transformation contained the selectable marker Zeocin, which is bifunctional in both *Pichia *and *Escherchia coli*, the 5'GAP promoter and the 3' AOX TT transcription termination sequences. The Pfx DNA polymerase, T4 DNA ligase, PCR purification kit and Midi-Prep Kit were purchased from Promega.

### Construction of TPL expression vector

The cDNA sequence encoding the TPL was previously isolated and cloned into the pGEM-T Easy vector in our laboratory by Fendri et *al *(2006).

Using the TPL cDNA as template, a DNA fragment of 1353 pb was amplified by PCR with sens (5'-GATCGAATTCTCTGAAGTTTGCTATGAC-3') and antisense (5'-GATCGAATTCTTAGCAAGCAGTAAGGGT-3') primers introducing EcoRI sites (underlined) immediately upstream of the first codon (TCT) and downstream of the stop codon.

The PCR product was then cloned into the PCR-Blunt-Topo vector using the PCR-Blunt-Topo cloning kit according to the manufactures's protocol (Invitrogen). Protoplasts of *E.coli *DH5α were transformed with the ligation mixture. The presence of the expected insert in the resulting plasmids was determined by restriction analysis.

The resulting recombinant vector PCR-Blunt-Topo-TPL was digested with the restriction enzyme EcoRI. The EcoRI-digested insert was purified and inserted into pGAPZαA *P.pastoris *transfert vector downstream of the GAP constitutive promoter as described by Sias [31].

The consequent plasmid (pGAPZαA-TPL, 4512pb) was transformed into *E. coli *DH5α by the chemical method [32] and the transformed clones were selected on Luria-Bertani (LB) plates containing 25 μg/ml Zeocin.

The recombinant plasmids were then insolated using the Midi-prep purification system. The gene coding for TPL with the α-factor was sequenced, proving that no mutation occurred during the PCR. Experiments were performed 3 times with different clones.

### Transformation of *P.pastoris *and screening of TPL transformants

Electrocompetent X33 cells, prepared using standard methods [33], was transformed with 10 μg of BspHI-linearized pGAPZαA-TPL by electroporation according to Invitrogen manual. The recombinant yeast clones were selected on YPDS plates containing 100 μg/ml Zeocin.

The colonies were subsequently screened by PCR using as template the genomic DNA extracted from different transformants and the pGAPZαA universal primers (pGAP Forward and AOX1 primers) to confirm the integration of the TPL DNA into the yeast genome.

Positive transformants were grown in 250 ml Erlenmeyer flasks containing 50 ml YPD medium with 100 μg/ml Zeocin, at 30°C under shaking at 150 rpm. The time-course of rTPL secretion into the culture medium was determined using various clones.

### Real-time PCR

The ICycler (Biorad) was used for Q-PCR amplification and detection. Q-PCR was prepared in 25 μl reaction mixture. Each reaction well contains 5 μl of template DNA (100 ng), 12.5 μl of SensiMix dT, 0.5 μl of SYBR Green I solution, 4 mM MgCl_2 _and 10 mM of forward (5'-GCAATAGGACATCTTGACTTT-3') and reverse (5'-ATCTGCATAGTGACCCATGTT-3') primers to generate an amplicon of 276 pb. Serial 10-fold dilutions of plasmid DNA (pGAPZαA/TPL) were conduced to establish the standard curve. The negative control (without DNA template) was included in experimental runs. The Q-PCR cycling program was 10 min at 95°C for activation of the hot-start enzyme, followed by 40 cycles of denaturation at 95°C for 15s, annealing at 52°C for 30s and elongation at 72°C for 30s. Melting curves analysis was performed after completed Q-PCR collecting fluorescence between 60 and 95°C at 1°C increments.

### Production and purification of rTPL in *P.pastoris*

The clone showing the most important activity level was selected to produce the recombinant protein. It was then pre-grown at 30° in 250 ml shake flasks containing 50 ml YPD medium for 24 h to an OD_600 _of 6. This cell culture was further used to inoculate five 500 ml shake flasks containing 100 ml YPD medium without Zeocin.

The culture was started at an optical density of 1 to establish reproducible cells culture conditions. The production of rTPL was conducted at 30°C for 4 days with shaking at 150 rpm.

Five hundred milliliters of culture medium were collected after fermentation and used to purify the recombinant enzyme by performing a five-step purification protocol comprising the following steps: an ammonium sulfate precipitation; anion exchange chromatography on DEAE-Cellulose; filtration on Sephacryl S200; anion exchange chromatography on Q-Sepharose FF and a filtration on Sephadex G100. Native TPL was purified according to the protocol established by Sayari et al [8].

### SDS-PAGE

Sodium dodecyl sulfate polyacrylamide gel electrophpresis (SDS-PAGE) was performed with a 12% polyacrylamide gel as described by Laemmli [34]. After electrophoresis, proteins were strained with coomassie brilliant blue R-250.

### Amino acid sequensing

The N-Terminal sequence of the recombinant enzyme were determined by automated Edman degradation, using an Applied Biosytems Procise 492 protein sequencer equipped with the 140 C HPLC system [35].

### Lipase activity determination

The lipase activity was measured titrimetically at pH 8.5 and 37°C with a pH-Stat using tributyrin (0.25 ml) in 30 ml of 2 mM Tris-HCl, pH 8.5 150 mM NaCl or olive oil emulsion in presence of 4 mM NaDC and Turkey colipase [36]. One lipase unit corresponds to 1 μmol of fatty acid liberated per min.

### Determination of protein concentration

Protein concentration was determined as described by Bradford [37] using BSA (E ^1%^_1 cm _= 6.7) as reference.

### Effect of temperature and pH on enzyme stability and activity

Optimal pH and temperature were determined examining the lipase activity at different temperature and pH by pH-Stat assay using olive oil as substrate. pH stability was determined by incubating lipase solution for 30 min at different pH at 4°C. Thermostability was determined by pre-incubating the enzyme at temperatures ranging from 37° to 60°C. The lipase activity was measured after centrifugation under standard conditions.

### Effect of bile salts and calcium ions on the enzyme activity

In order to study the bile salt effect on the rTPL and nTPL activities, lipase activity was measured using emulsified olive oil as substrate at 37°C and pH 8.5 in the presence of increasing concentration of sodium deoxycholate (NaDC) in both the presence and the absence of an excess of colipase.

To study the calcium dependence of the two enzymes, lipase activity was measured using emulsified olive oil as substrate at 37°C and pH 8.5 in the presence of an excess of colipase, 4 mM NaDC and an increasing concentrations of CaCl_2_.

## Competing interests

The authors declare that they have no competing interests.

## Authors' contributions

MBA and YBA carried out the studies, analyzed the data and drafted the manuscript. AK helped technically and with the analysis of the data. AF participated in the study design and helped to draft the manuscript. YG helped with the discussion of the data and the correction of the manuscript. All authors have read and approved the final manuscript.

## References

[B1] Schmid RD Verger R Lipases interfacial enzymes with attractive applications Angewandte Chem. Int. Ed 1998 37 1608 1633 10.1002/(SICI)1521-3773(19980703)37:12<1608::AID-ANIE1608>3.0.CO;2-V 29711530

[B2] Winkler FK D'arcy A Hunziker W Structure of human pancreatic lipase Nature 1990 343 771 774 10.1038/343771a0 2106079

[B3] Van Tilbeurgh H Egloff MP Martinez C Rugani N Verger R Cambillau C Interfacial activation of the lipase-procolipase complex by mixed micelles revealed by X-ray crystallography Nature 1993 362 814 820 10.1038/362814a0 8479519

[B4] Ollis DL Cheah E Cygler M Dijkstra B Frolow F Franken SM Harel M Remington SJ Silman I Schrag J The α/β hydrolase fold Prot. Eng 1992 5 197 211 10.1093/protein/5.3.197 1409539

[B5] Van Tilbeurgh H Sarda L Verger L Cambillau C Structure of the pancreatic lipase-procolipase complex Nature 1992 359 159 162 10.1038/359159a0 1522902

[B6] Withers-Martinez C Carrière F Verger R Bourgeois D Cambillau C A pancreatic lipase with a phospholipase A1 activity: crystal structure of a chimeric pancreatic lipase-related protein 2 from guinea pig Structure 1996 4 1363 1374 10.1016/S0969-2126(96)00143-8 8939760

[B7] Fendri A Frikha F Miled N Gargouri Y Cloning and molecular modelling of turkey pancreatic lipase: structural explanation of the increased interaction power with lipidic interface Biochimie 2006 88 1401 1407 10.1016/j.biochi.2006.05.022 16828950

[B8] Sayari A Mejdoub H Gargouri Y Characterization of turkey pancreatic lipase Biochimie 2000 82 153 159 10.1016/S0300-9084(00)00379-5 10727771

[B9] Fendri A Sayari A Gargouri Y Kinetic properties of turkey pancreatic lipase: a comparative study with emulsified tributyrin and monomolecular dicaprin Chirality 2005 17 57 62 10.1002/chir.20097 15549712

[B10] Gargouri Y Pieroni G Lowe PA Sarda L Verger R Human gastric lipase. The effect of amphiphiles Eur. J. Biochem 1986 156 305 310 10.1111/j.1432-1033.1986.tb09583.x 3699017

[B11] Gargouri Y Bensalah A Douchet I Verger R Kinetic behaviour of five pancreatic lipases using emulsion and monomolecular films of synthetic glycerides Biochem. Biophys. Acta 1995 1257 223 229 10.1016/0005-2760(95)00071-j 7647098

[B12] Ben Bacha A Fendri A Gargouri Y Mejdoub H Miled N Proteolytic cleavage of ostrich and turkey pancreatic lipases: production of an active N-terminal domain Pancreas 2007 35 55 61 10.1097/mpa.0b013e31811f450f 17895836

[B13] Bousset-Risso M Bonicel J Rovery M Limited proteolysis of porcine pancreatic lipase: Lability of the Phe 335-Ala 336 bond towards chymotrypsin FEBS lett 1985 182 323 326 10.1016/0014-5793(85)80325-2 3979556

[B14] Abousalham A Chaillan C Kerfelee B Uncoupling of catalysis and colipase binding in pancreatic lipase by limited proteolysis Prot. Eng 1992 5 105 111 10.1093/protein/5.1.105 1631040

[B15] Gellissen G Heterologous protein production in methylotrophic yeasts Appl. Microbiol. Biotechnol 2000 54 741 750 10.1007/s002530000464 11152064

[B16] Cereghino JL Cregg JM Heterologous protein expression in the methylotrophic yeast *Pichia pastoris* FEMS Microbiol. Rev 2000 24 45 66 10.1111/j.1574-6976.2000.tb00532.x 10640598

[B17] Waterham HR Digan ME Koutz PJ Lair SV Cregg JM Isolation of the *Pichia pastoris *glyceraldehyde-3-phosphate dehydrogenase gene and regulation and use of its promoter Gene 1997 186 37 44 10.1016/S0378-1119(96)00675-0 9047342

[B18] Mattanovich D Gasser B Hohenblum H Sauer M Stress in recombinant protein producing yeasts Journal of Biotechnology 2004 113 121 135 10.1016/j.jbiotec.2004.04.035 15380652

[B19] Sato R Matsumoto T Hidaka N Imai Y Abe K Takahashi S Yamada R Kera Y Cloning and expression of carp acetylcholinesterase gene in *Pichia pastoris *and characterization of the recombinant enzyme Protein Expression and Purification 2009 64 205 212 10.1016/j.pep.2008.12.003 19121395

[B20] Sebban-Kreuzer C Deprez-Beauclair P Berton A Crenon I High-level expression of nonglycosylated human pancreatic lipase-related protein 2 in *Pichia pastoris* Protein Expression and Purification 2006 49 284 291 10.1016/j.pep.2006.06.001 16861001

[B21] Aloulou A Grandval P De Caro J De Caro A Carrière F Constitutive expression of human pancreatic lipase-related protein 1 in *Pichia pastoris* Protein Expression and Purification 2006 47 415 421 10.1016/j.pep.2006.01.004 16481202

[B22] Murasugi1 A Asami Y Mera-Kikuchi Y Production of Recombinant Human Bile Salt-Stimulated Lipase in *Pichia pastoris* Protein Expression and Purification 2001 23 282 288 10.1006/prep.2001.1509 11676603

[B23] Yu M Lange S Richter S Tan T Schmid RD High-level expression of extracellular lipase Lip2 from *Yarrowia lipolytica *in *Pichia pastoris *and its purification and characterization Protein Expression and Purification 2007 53 255 263 10.1016/j.pep.2006.10.018 17321147

[B24] Tang SJ Shaw JF Sun KH Sun GH Chang TY Lin CK Lo YC Lee GC Recombinant expression and characterization of the *Candida rugosa *lip4 lipase in *Pichia pastoris*: comparison of glycosylation, activity, and stability Arch. Biochem. Biophys 2001 387 93 98 10.1006/abbi.2000.2235 11368188

[B25] Richter S Nieveler J Schulze H Bachmann TT Schmid RD High yield production of a mutant *Nippostrongylus brasiliensis *Acetylcholinesterase in *Pichia pastoris *and its purification Biotechnol. Bioeng 2005 93 1017 1022 10.1002/bit.20705 16302258

[B26] Gargouri Y Julien R Bois AG Verger R Sarda L Studies on the detergent inhibition of pancreatic lipase activity J. Lipid. Res 1983 24 1336 1342 6644184

[B27] Gargouri Y Pièroni G Lowe PA Sarda L Verger R Human gastric lipase: the effect of amphiphiles Eur. J. Biochem 1985 156 305 310 10.1111/j.1432-1033.1986.tb09583.x 3699017

[B28] Ben Bacha A Gargouri Y Ben Ali Y Miled N Reinbolt J Mejdoub H Purification and biochemical characterization of ostrich pancreatic lipase Enzyme and Microbial Technology 2005 37 309 317 10.1016/j.enzmictec.2004.07.022

[B29] Benzonana G On the role of Ca2+ during the hydrolysis of insoluble triglycerides by pancreatic lipase in the presence of bile salts Biochim. Biophys. Acta 1968 151 137 146 5689112 10.1016/0005-2744(68)90168-x

[B30] Horchani H Sabrina L Régine L Sayari A Gargouri Y Verger R Heterologous expression and N-terminal His-tagging processes affect the catalytic properties of staphylococcal lipases: A monolayer study Journal of Colloid and Interface Science 2010 350 586 594 10.1016/j.jcis.2010.07.021 20684959

[B31] Sias B Ferrato F Grandval P Lafont D Boullanger P De Caro A Leboeuf B Verger RF Carriere, Human pancreatic lipase-related protein 2 is a galactolipase Biochemistry 2004 43 10138 10148 10.1021/bi049818d 15287741

[B32] Sambrook J Fritsch EF Maniatis T Molecular Cloning: A Laboratoty Manual 1989 Cold Spring Harbor Laboratory Press, New York

[B33] Cregg JM Russell KA Transformation Methods Mol. Biol 1998 103 27 39 9680631 10.1385/0-89603-421-6:27

[B34] Laemmli UK Cleavage of structural proteins during the assembly of the head of bacteriophage T4 Nature 1970 227 680 685 10.1038/227680a0 5432063

[B35] Hewick RM Hunkapiller MW Hood LE Dreyer WJ A gas-liquid solid phase peptide and protein sequenator J.Biol. Chem 1981 256 7990 7997 7263636

[B36] Rathelot J Julien R Canioni P Coeroli C Sarda L Studies on the effect of bile salt and colipase on enzymatic lipolysis. Improved method for the determination of pancreatic lipase and colipase Biochimie 1975 57 1117 1122 10.1016/S0300-9084(76)80572-X 1222120

[B37] Bradford MM A rapid and sensitive method for the quantitation of protein utilizing the principle of protein-dye binding Anal. Biochem 1976 72 248 254 10.1016/0003-2697(76)90527-3 942051

